# Amelioration of pancreatic fat accumulation in Japanese type 2 diabetes patients treated with sodium‐glucose cotransporter 2 inhibitors: a retrospective study

**DOI:** 10.1002/osp4.482

**Published:** 2021-02-08

**Authors:** Tomomi Horii, Junji Kozawa, Shingo Fujita, Yoshiya Hosokawa, Takekazu Kimura, Yukari Fujita, Ayumi Tokunaga, Kenji Fukui, Iichiro Shimomura

**Affiliations:** ^1^ Department of Metabolic Medicine Graduate School of Medicine Osaka University Suita Japan; ^2^ Department of Diabetes Care Medicine Graduate School of Medicine Osaka University Suita Japan; ^3^ Department of Community Medicine Graduate School of Medicine Osaka University Suita Japan

**Keywords:** pancreatic fat, SGLT‐2 inhibitor, terms: type 2 diabetes

## Abstract

**Background:**

Prior reports have suggested that pancreatic fat is related to type 2 diabetes. Sodium‐glucose co‐transporter‐2 (SGLT‐2) inhibitors are expected to reduce ectopic fat accumulation.

**Aim:**

This study assessed the effect of SGLT‐2 inhibitors on pancreatic and liver fat accumulations in patients with type 2 diabetes.

**Materials and Methods:**

Retrospective analyses of indices of pancreatic and liver fat accumulations were conducted in 22 type 2 diabetes outpatients who were receiving SGLT‐2 inhibitors for more than 12 weeks. The differences between the pancreatic (P) or liver (L) and splenic (S) computed tomography values were evaluated.

**Results:**

Fatty pancreas was defined as P−S < −8 Hounsfield Unit (HU), and the number of patients with fatty pancreas was 11 (50%). Fatty pancreas significantly improved after SGLT‐2 inhibitor use (median, −20.8; IQR, −34.8 to −14.3 HU vs. median, −14.6; IQR, −29.5 to −7.8 HU; *p* = 0.041). Fatty liver was defined as L−S ≤ 3.9 HU, and the number of patients with fatty liver was 11 (50%). Fatty liver significantly improved after SGLT‐2 inhibitor use (median, −4.3; IQR, −23.0 to 3.0 HU vs. median, −0.7; IQR, −5.2 to 6.3 HU; *p* = 0.016).

**Conclusion:**

Pancreatic fat and liver fat accumulations might be reduced after treatment with SGLT‐2 inhibitors in type 2 diabetes patients with intense cumulative fat depositions in these organs.

## 1 INTRODUCTION

Ectopic fat accumulation is the deposition of excess lipids in various organs.^1^ Nonalcoholic fatty liver disease (NAFLD) is the form of liver disease derived from ectopic fat deposition, and insulin resistance due to NAFLD is the important risk factor for the incidence of type 2 diabetes mellitus.^2^ Ectopic fat accumulation is also found in pancreas in addition to other organs.^3^, ^4^ The role of fat accumulation in the pancreas in the pathogenesis of type 2 diabetes has been discussed.^3^ Pancreatic fat accumulation measured by computed tomography (CT) scans was strongly associated with the longitudinal decrease in endogenous insulin‐secreting capacity.^5^ Histological pancreatic fatty infiltration had a correlation with glucose intolerance within 1 year of pancreatectomy in preoperative nondiabetic patients.^4^ Recently, it has been elucidated that this fatty infiltration is associated with islet inflammation.^6^ That report suggests that pancreatic fat is closely related to the pathophysiology of type 2 diabetes mellitus and glycemic control.

Sodium‐glucose co‐transporter‐2 (SGLT‐2) inhibitors are oral hypoglycemic drugs that prevent the reabsorption of glucose in the proximal renal tubules and enhance urinary glucose excretion, resulting in a decrease in blood glucose levels.^7^ In addition to the hypoglycemic action, SGLT‐2 inhibitors exert reducing effects on body weight and visceral adipose tissue.^8^ In patients with type 2 diabetes and NAFLD, the administration of dapagliflozin improved liver steatosis and attenuated liver fibrosis.^9^ Thus, SGLT‐2 inhibitors are expected to reduce ectopic fat accumulation in various organs.

In this study, pancreatic and liver fat accumulations were assessed in patients with type 2 diabetes before and after treatment with SGLT‐2 inhibitors, then the effect of SGLT‐2 inhibitors on these fat accumulations were evaluated.

## 2 METHODS

### 2.1 Patients

This is a retrospective study in which 22 outpatients with type 2 diabetes at the Department of Metabolic Medicine, Osaka University Hospital between April 2014 and April 2020, and had been treated with SGLT‐2 inhibitors for more than 12 weeks. Abdominal follow‐up CT with unenhanced phases was done for underlying diseases before starting SGLT‐2 inhibitors and at least 12 weeks after starting them. At the time of starting SGLT‐2 inhibitors, 21 patients received oral antidiabetic agents other than SGLT‐2 inhibitors and/or insulin.

### 2.2 Clinical parameters

The following data were obtained from medical records: age, sex, HbA1c, plasma glucose (PG), total cholesterol, triglycerides, high‐density lipoprotein cholesterol (HDL‐C), low‐density lipoprotein cholesterol (LDL‐C), aspartate aminotransferase (AST), alanine aminotransferase (ALT), γ‐glutamyl transpeptidase (γ‐GTP), estimated glomerular filtration rate (eGFR), uric acid, serum creatinine concentration, urea nitrogen, albumin, hematocrit, and fibrosis 4 (FIB4) index, body weight and body mass index (BMI). The eGFR was calculated using the equation of the Japanese Society of Nephrology: eGFR (ml/min/1.73 m^2^) = 194 × Cr^−1.094^ × age^−0.287^ (×0.739 for women). The FIB4 index was defined as age × AST (platelet count [10^9^/L]) × √AST).

### 2.3 CT protocols

The differences between the pancreatic (P) and splenic (S) CT values (P−S), the differences between the liver (L) and splenic CT values (L−S), visceral fat area (VFA) (cm^2^), subcutaneous fat area (SFA) (cm^2^), and the ratio of VFA to SFA (V/S ratio) were measured. Two patients whose VFA and SFA were considered to be inaccurate were excluded from the evaluation of these parameters because one patient was not imaged on the entire trunk, and VFA and SFA of the other patient were affected by artifacts of the wrist joint located on the umbilical region. To evaluate P−S and L−S, the regions of interest (ROIs) were specified using areas of 1.0 cm^2^ in sites that did not contain major vessels and measured the Hounsfield units (HU; CT value) from one ROI each. A pancreatic CT value is defined as the mean CT value in three different pancreatic parts: head, body and tail. A liver CT value is defined as the mean CT value in three different liver parts: anterior and posterior right lobe and left lobe. A splenic CT value is defined as the mean CT value in three different splenic parts: upper, middle and lower. From the aforementioned pancreatic CT value and splenic CT value, indices of pancreatic fat content were defined as P−S, which were calculated as previously shown.^10^ Similarly, indices of liver fat content were defined as L−S, which were calculated as previously shown.^11^ The CT values were analyzed using the software program Aquarius Net Viewer Version 4.4 (TeraRecon, Inc.). The VFA, SFA and V/S were measured using SYNAPSE VINCENT image analysis system (Fujifilm, Inc.).

### 2.4 Statistical analysis

Normally distributed data are presented as the means ± SD, and nonnormally distributed data are presented as the medians and interquartile ranges. Changes in continuous clinical parameters before and after the treatment period were tested using a paired *t* test, and changes in P−S and L−S before and after the treatment period were tested using the Wilcoxon signed‐rank test for nonnormally distributed values. *p* values < 0.05 were regarded as statistically significant. All statistical analyses were performed with JMP Pro 14 software (SAS Institute Inc.).

## 3 RESULTS

Table 1 shows the baseline characteristics of patients and the underlying diseases requiring abdominal follow‐up CT in addition to the changes in the clinical parameters at an average of 33 ± 14 weeks after compared with those at baseline. The kinds of SGLT‐2 inhibitors were canagliflozin in seven patients, empagliflozin in six, dapagliflozin in five, ipragliflozin in three, and luseogliflozin in one.

**TABLE 1:** Clinical Characteristics at baseline and after administration of SGLT‐2 inhibitors in 22 patients with type 2 diabetes

	Baseline	33 ± 14 weeks	*p* values
Age, y	64.9 ± 10.4		
Male/female	16/6		
HbA1c, mmol/mol	64.3 ± 10.4	54.9 ± 8.1	<0.0001
HbA1c, %	8.0 ± 0.9	7.2 ± 0.7	<0.0001
Plasma glucose, mmol/L	9.1 ± 2.8	7.5 ± 2.5	0.0009
Total cholesterol, mmol/L (*n* = 17)	4.5 ± 0.7	4.6 ± 0.7	0.54
Triglycerides, mmol/L (*n* = 20)	1.8 ± 1.2	1.8 ± 1.3	0.31
HDL‐C, mmol/L (*n* = 20)	1.2 ± 0.4	1.3 ± 0.3	0.022
LDL‐C, mmol/L (*n* = 18)	2.5 ± 0.9	2.7 ± 0.7	0.68
AST, IU/L	38.2 ± 17.2	33.6 ± 16.0	0.081
ALT, IU/L	38.9 ± 20.5	34.7 ± 20.0	0.18
γ GTP, IU/L (*n* = 20)	74.2 ± 76.8	56.5 ± 67.7	0.0003
eGFR, ml/min/1.73 m^2^ (*n* = 21)	64.1 ± 19.2	59.7 ± 18.6	0.0058
Uric acid, μmol/L (*n* = 19)	311.5 ± 74.4	293.8 ± 67.7	0.11
Creatinine, μmol/L (*n* = 21)	83.8 ± 26.7	88.5 ± 28.5	0.0026
Urea nitrogen, mmol/L (*n* = 18)	5.9 ± 1.7	6.6 ± 1.9	0.11
Albumin, g/L (*n* = 14)	40.0 ± 3.2	41.2 ± 3.7	0.68
Hematocrit,/L (*n* = 21)	0.42 ± 0.05	0.44 ± 0.05	0.0015
FIB4 index (*n* = 19)	3.0 ± 3.0	3.0 ± 4.1	0.40
Body weight, kg	78.6 ± 23.0	75.6 ± 23.7	<0.0001
BMI, kg/m^2^	29.2 ± 6.6	28.1 ± 6.9	<0.0001
P−S	−8.0 (−20.9–−5.9)	−10.7 (−16.2–−5.2)	0.86
L−S	5.4 (−5.0–13.3)	6.0 (−1.5–10.3)	0.60
VFA, cm^2^ (*n* = 20)	163.2 ± 69.6	148.9 ± 69.6	0.0042
SFA, cm^2^ (*n* = 20)	182.9 ± 75.4	171.4 ± 82.6	0.035
V/S (*n* = 20)	0.97 ± 0.4	0.96 ± 0.5	0.74
Underlying disease	Simple obesity: 1, fatty liver disease: 1, thoracic or abdominal aortic aneurysm: 5, post conservative treatment of aortic dissection: 1, primary aldosteronism: 1, IPMN: 2, relapsing polychondritis: 1, multiple myeloma: 1, autoimmune pancreatitis: 1, postoperation for cancer (hepatocellular carcinoma: 5, pancreatic cancer: 1, colorectal cancer: 2)		

*Note*: Values are mean ± SD.

Abbreviations: ALT, alanine amino transferase; AST, aspartate amino transferase; BMI, body mass index; CPR, C‐peptide; FIB4 index, age × AST (platelet count (109/L)) × √AST); GA, glycoalbumin; HDL‐C, high density lipoprotein cholesterol; IPMN, Intraductal papillary mucinous neoplasm; IRI, immunoreactive insulin; LDL‐C, low density lipoprotein cholesterol; L−S, the differences between the liver (L) and splenic (S) CT values; P−S, the differences between the pancreatic (P) and splenic (S) CT values; SFA, subcutaneous fat area; VFA, visceral fat area; V/S, the ratio of VFA to SFA; γGTP, γ‐glutamyl transpeptidase.

The changes in HbA1c (8.0 ± 0.9 to 7.2 ± 0.7%; *p* < 0.0001) and PG levels (9.1 ± 2.8 to 7.5 ± 2.5 mmol/L; *p* < 0.0001) decreased significantly compared with those at baseline. The change in γ‐GTP levels showed significant decrease (74.2 ± 76.8 to 56.5 ± 67.7 IU/L; *p* = 0.0003). There was a slight decrease in eGFR (64.1 ± 19.2 to 59.7 ± 18.6 mL/min/1.73 m^2^; *p* = 0.0058) and increase in creatinine (83.8 ± 26.7 to 88.5 ± 28.5 μmol/L; *p* = 0.0026). The body weight (78.6 ± 23.0 to 75.6 ± 23.7 kg; *p* < 0.0001), BMI (29.2 ± 6.6 to 28.1 ± 6.9 kg/m^2^; *p* < 0.0001), the VFA (163.2 ± 69.6 to 148.9 ± 69.6 cm^2^; *p* = 0.0042) and the SFA (182.9 ± 75.4 to 171.4 ± 82.6 cm^2^; *p* = 0.035) showed significant decrease.

There was no significant difference in P−S before and after taking SGLT‐2 inhibitors (median, −8.0; IQR, −20.9 to −5.9 HU vs. median, −10.7; IQR, −16.2 to −5.2 HU; *p* = 0.86; Table 1). Referring to the scatterplots and regression lines between histologic pancreatic fat fraction and CT attenuation indices that Kim SY et al. reported,^10^ P−S < −8 seemed to reflect severe pancreatic fat deposition because this is equivalent to CT attenuation indices of less than lower interquartile and pancreatic fat fraction of more than 10% in patients who had undergone pancreatic resection. If P−S < −8 was classified as fatty pancreas, the incidence of fatty pancreas was 11 out of 22 patients. In these patients, there was a significant increase in P−S after SGLT‐2 inhibitor use compared with the values before taking SGLT‐2 inhibitors (median, −20.8; IQR, −34.8 to −14.3 HU vs. median, −14.6; IQR, −29.5 to −7.8 HU; *p* = 0.041; Figure S1). In addition, the analysis excluding one person who had the past history of pancreatic tumor and underwent pancreatoduodenectomy showed a further significant improvement (*p* = 0.0049). There were significant decreases in body weight (70.8 ± 17.9 to 68.0 ± 19.6 kg; *p* = 0.0054) and BMI (27.5 ± 5.1 to 26.4 ± 5.8 kg/m^2^; *p* = 0.0010) in addition to HbA1c (8.0 ± 1.0 to 7.2 ± 0.7%; *p* = 0.027), PG levels (9.7 ± 3.4 to 7.6 ± 3.0 mmol/L; *p* = 0.046) and VFA (155.5 ± 51.7 to 137.4 ± 58.8 cm^2^; *p* = 0.021; Table 2). However, there was no significant correlation between the increase of P−S and the reduction in body weight (*r* = 0.11, *p* = 0.74), BMI (*r* = 0.17, *p* = 0.62) or VFA (*r* = −0.12, *p* = 0.73).

**TABLE 2:** The changes in indices of fat content and physical parameters between baseline and after the administration of SGLT‐2 inhibitors in 11 patients with P−S < −8 and 11 patients with L−S ≤ 3.9

	Baseline	36 ± 15 weeks	*p* values
P−S	−20.8 (−34.8–−14.3)	−14.6 (−29.5–−7.8)	0.041
HbA1c, mmol/mol	64.9 ± 11.4	55.6 ± 7.6	0.027
HbA1c, %	8.0 ± 1.0	7.2 ± 0.7	0.027
Plasma glucose, mmol/L	9.7 ± 3.4	7.6 ± 3.0	0.046
VFA, cm^2^ (*n* = 10)	155.5 ± 51.7	137.4 ± 58.4	0.021
SFA, cm^2^ (*n* = 10)	164.6 ± 55.8	144.1 ± 53.3	0.014
V/S (*n* = 10)	1.1 ± 0.5	1.0 ± 0.5	0.56
Body weight, kg	70.8 ± 17.9	68.0 ± 19.6	0.0054
BMI, kg/m^2^	27.5 ± 5.1	26.4 ± 5.8	0.0010

	Baseline	32 ± 12 weeks	*p* values
L−S	−4.3 (−23.0–3.0)	−0.7 (−5.2–6.3)	0.016
HbA1c, mmol/mol	60.9 ± 12.2	51.9 ± 5.1	0.034
HbA1c, %	7.7 ± 1.1	6.9 ± 0.4	0.034
Plasma glucose, mmol/L	8.4 ± 1.8	7.3 ± 2.2	0.037
AST, IU/L	48.5 ± 17.9	36.8 ± 16.3	0.064
ALT, IU/L	49.3 ± 20.0	36.8 ± 16.2	0.080
FIB4 index	2.8 ± 1.7	2.3 ± 1.5	0.039
VFA, cm^2^	198.6 ± 58.6	174.0 ± 64.8	0.040
SFA, cm^2^	207.9 ± 89.3	199.9 ± 96.0	0.43
V/S	1.1 ± 0.4	1.0 ± 0.3	0.22
Body weight, kg	81.8 ± 27.5	78.7 ± 27.9	0.0093
BMI, kg/m^2^	30.1 ± 8.0	29.0 ± 8.2	0.0093

*Note*: Values are mean ± SD or median (25th–75th percentiles).

Abbreviations: BMI, body mass index; L−S, the differences between the liver (L) and splenic (S) CT values; P−S, the differences between the pancreatic (P) and splenic (S) CT values; SFA, subcutaneous fat area; VFA, visceral fat area; V/S, the ratio of VFA to SFA.

There was no significant difference in the L−S before and after treatment with SGLT‐2 inhibitors (median, 5.4; IQR, −5.0 to 13.3 HU vs. median, 6.0; IQR, −1.5 to 10.3 HU; *p* = 0.60; Table 1). Ahn Y et al.^12^ reported that cutoff values of 3.9 for L−S provided 90% specificity for diagnosis of hepatic steatosis. When fatty liver was defined as L−S≤3.9, the incidence of fatty liver was 11 out of 22 patients. In these patients, there was a significant increase in L−S after SGLT‐2 inhibitor use compared with the value before taking SGLT‐2 inhibitors (median, −4.3; IQR, −23.0 to 3.0 HU vs. median, −0.7; IQR, −5.2 to 6.3 HU; *p* = 0.016; Figure S2). In addition, the analysis excluding three persons who had the past history of hepatocellular carcinoma also showed a significant improvement (*p* = 0.039). There were significant decreases in body weight (81.8 ± 27.5 to 78.7 ± 27.9 kg; *p* = 0.0093) and BMI (30.1 ± 8.0 to 29.0 ± 8.2 kg/m^2^; *p* = 0.0093) in addition to HbA1c (7.7 ± 1.1 to 6.9 ± 0.4%; *p* = 0.034), PG levels (8.4 ± 1.8 to 7.3 ± 2.2 mmol/L; *p* = 0.037) and VFA (198.6 ± 58.6 to 174.0 ± 64.8 cm^2^; *p* = 0.040; Table 2). There was no significant correlation between the increase of L−S and the reduction in body weight (*r* = −0.02, *p* = 0.95), BMI (*r* = −0.11, *p* = 0.75) or VFA (*r* = −0.31, *p* = 0.37).

The total number of patients with fatty pancreas or fatty liver before treatment was 17. Concurrence of fatty pancreas and fatty liver was found in five patients. In other words, not all 11 patients with fatty pancreas overlapped with 11 patients with fatty liver.

## 4 DISCUSSION

The present study showed a possible amelioration of pancreatic fat as well as liver fat accumulations with SGLT‐2 inhibitor use in cases with intense cumulative fat depositions in these organs, with improvements in various clinical parameters. This is the first study that showed a possible effect of SGLT‐2 inhibitor on pancreatic fat accumulation.

One of the possible mechanisms by which SGLT‐2 inhibitors affect pancreatic and liver fat might be assumed to be associated with a reduction in body weight and visceral fat. The prior study in patients with a BMI above 30 kg/m^2^ reported that after administration of canagliflozin, approximately 70% of the reduction in body weight was due to a decrease in fat mass, and the observed decrease in fat mass was due to a 13.7% reduction in VFA.^13^ In this study, body weight, BMI and VFA significantly decreased. However, there were no significant correlations between the reduction in these parameters and the reduction in pancreatic or liver fat in patients with intense cumulative fat depositions in these organs. Honka et al. reported that although the decrease in pancreatic fat was detected after bariatric surgery, any association was not found with the degree of the change in body weight or visceral fat mass.^14^ Patel et al. reported that a significant reduction in BMI of at least 5% did not lead to a significant decrease in pancreatic fat.^15^ As to liver fat, there are several reports that analyze the association between the decrease in body weight and the decrease in liver fat in patients with SGLT‐2 inhibitors use; Takase et al. reported that the change in BMI and VFA under the treatment with ipragliflozin tended to be associated with the change in fatty liver index.^16^ Patel et al. also reported that a reduction in BMI of at least 5% is associated with significant decrease in liver fat and volume in patients with nonalcoholic steatohepatitis.^15^ On the other hand, Kuchay et al. reported that liver fat reduction under the treatment with empagliflozin is irrespective of body weight reduction.^17^ These are still controversial, however, considering these reports and the present study, the reduction in pancreatic and liver fat may be mediated by factors other than or additional to the reduction in body weight or VFA after the treatment with SGLT‐2 inhibitors.

Another mechanism may be associated with a reduction in insulin levels, although this could not be affirmed in the study due to insufficient data. Insulin‐signaling pathways play a significant role in adipogenesis.^18^ Pancreatic islets transplanted into portal vein can induce focal hepatic steatosis in type 1 diabetes recipient,^19^ which indicates that insulin secretion promotes local fat deposition. The higher local insulin concentration may promote fat accumulation also in pancreas, as was discussed.^20^ SGLT‐2 inhibitors promote glycosuria and reduce blood glucose levels, resulting in the reduction of insulin levels systemically^7^ and, maybe, also in paracrine manner from β cell to surrounding pancreatic cells. Therefore, the amelioration of pancreatic fat may be partly attributed to the reduction in insulin levels.

Previous reports showed negative effect of pancreatic fat accumulation on insulin‐secreting capacity in patients with various degrees of glucose intolerance^21^, ^22^ and in patients with type 2 diabetes.^5^ Improvement of pancreatic fat accumulation under treatment with SGLT‐2 inhibitors may contribute to the long‐term retention of insulin‐secreting capacity. Further prospective studies would be needed to confirm it.

Not all 11 patients with fatty pancreas overlapped with 11 patients with fatty liver. This might be derived from the differences in etiology causing these ectopic fat accumulations. In this study, baseline P−S was not significantly associated with baseline L−S, and baseline L−S tended to be negatively correlated with BMI (*r* = −0.39, *p* = 0.069) and significantly correlated with AST (*r* = −0.65, *p* = 0.001), ALT (*r* = −0.45, *p* = 0.037), and VFA (*r* = −0.54, *p* = 0.0102), while baseline P−S was not correlated with these parameters. This means that pancreatic fat accumulation is less associated with obesity‐related parameters than liver fat. Some other reasons why patients with fatty pancreas were not identical to patients with fatty liver could be considered as follows; first, hepatic fat is mainly derived from intracellular fat deposition, while pancreatic fat is mainly derived from adipocytes infiltration,^23^ second, fat loss in pancreas seems to be independent of that in liver in patients with bariatric surgery, suggesting tissue‐specific mobilization of these ectopic fat stores,^24^ finally, pancreatic fat has a significant correlation with age,^24^, ^25^ while liver fat does not,^24^ which are consistent with the results of this study (data not shown).

There were several limitations in the present study. First, this is a retrospective observational study and the sample size was small. It can't be concluded that the results were solely derived from the effect of SGLT‐2 inhibitors. Second, histological confirmation of fat accumulation in pancreas and liver could not been done in these patients. However, P−S and L−S is strongly associated with histological pancreatic and liver fat fraction.^10^ Third, the effects of other diabetes therapy including insulin, sulfonylurea and glucagon‐like peptide‐1 receptor agonists on pancreatic fat and liver fat accumulations could not be excluded. Finally, all the patients had the underlying disease which could also affect these fat accumulations. In the future, a long‐term, large‐scale study would be needed to validate this finding and elucidate the clinical significance of the reduction in pancreatic fat on SGLT‐2 inhibitors.

In conclusion, the present study suggests that pancreatic fat and liver fat measured by abdominal CT scans might be ameliorated after treatment with SGLT‐2 inhibitors in type 2 diabetes patients with intense fat depositions in these organs. SGLT‐2 inhibitors might have the possibility that could prevent the development of pancreatic steatosis, leading to the preservation of β‐cell function and glycemic control.

## CONFLICT OF INTEREST

Junji Kozawa is a staff member of the endowed chair (Department of Diabetes Care Medicine) established by funds from Mitsubishi Tanabe Pharma Co.; AstraZeneca; Nippon Boehringer Ingelheim Co.; Taisho Pharmaceutical Co.; Ono Pharmaceutical Co.; MSD Co.; Keiseikai Hospital. Iichiro Shimomura has received lecture fees from Astellas Pharma, Inc.; MSD; Ono Pharmaceutical Co.; Kowa Pharmaceutical Co. Ltd.; Sanofi; Sanwa Kagaku Kenkyusho Co.; Takeda Pharmaceutical Co.; Mitsubishi Tanabe Pharma Co.; Eli Lilly Japan; Nippon Boehringer Ingelheim Co. and Novo Nordisk Pharma Ltd. and has received research funds from Astellas Pharma, Inc.; AstraZeneca; MSD; Ono Pharmaceutical Co.; Kyowa Kirin Co.; Kowa Company, Ltd.; Sanofi; Daiichi Sankyo Co.; Takeda Pharmaceutical Co.; Mitsubishi Tanabe Pharma Co.; Teijin Pharma Ltd.; Nippon Boehringer Ingelheim Co., Ltd; Novartis Pharmaceuticals Corp.; Novo Nordisk Pharma Ltd.; Taisho Pharmaceutical Co.; Sumitomo Dainippon Pharma Co.; Takeda Pharmaceutical Co.; Mitsubishi Tanabe Pharma Co.; Keiseikai Hospital; Gokeikai Osaka Kaisei Hospital; Midori Health Care Center; Suzuken Memorial Foundation; Japan Diabetes Foundation; Japan Foundation for Applied Enzymology; The Japan Diabetes Society and Terumo Co. Tomomi Horii, Shingo Fujita, Yoshiya Hosokawa, Takekazu Kimura, Yukari Fujita, Ayumi Tokunaga, and Kenji Fukui have nothing to declare.

## AUTHOR CONTRIBUTIONS

Tomomi Horii collected the data, analyzed them and wrote manuscript. Junji Kozawa, Shingo Fujita, Yoshiya Hosokawa, Takekazu Kimura, Yukari Fujita, Ayumi Tokunaga, Kenji Fukui, and Iichiro Shimomura contributed to the analysis and discussion. Junji Kozawa reviewed/edited the manuscript. Tomomi Horii is the guarantor of this work and, as such, had full access to all the data in the study and takes responsibility for the integrity of the data and the accuracy of data analysis. All authors read and approved the manuscript.

## Supporting information

Supplementary Material
