# Supplementary material for: Amelioration of pancreatic fat accumulation in Japanese type 2 diabetes patients treated with sodium‐glucose cotransporter 2 inhibitors: a retrospective study
Source: Obes Sci Pract. 2021 Feb 8;7(3):346–52. doi: 10.1002/osp4.482 (PMC8170578; doi:10.1002/osp4.482)
Supplement: Supplementary file 1 — Supplementary Material [file OSP4-7-346-s001.pdf]

Title: Amelioration of pancreatic fat accumulation in Japanese type 2 diabetes patients treated with sodium-glucose cotransporter 2 inhibitors: a retrospective study.

Tomomi Horii[1], Junji Kozawa[1,2], Shingo Fujita[1], Yoshiya Hosokawa[1], Takekazu Kimura[1], Yukari Fujita[1,3], Ayumi Tokunaga[1], Kenji Fukui[1], and Iichiro Shimomura[1]

Departments of [1]Metabolic Medicine, [2]Diabetes Care Medicine, and [3]Community Medicine, Graduate School of Medicine, Osaka University, Suita, Japan.

Corresponding author:
Junji Kozawa, M.D., Ph.D.
Department of Metabolic Medicine, Graduate School of Medicine, Osaka University.
2-2-B5 Yamadaoka, Suita 565-0871, Japan.
Tel.: +81-6-6879-3732, Fax: +81-6-6879-3739.
E-mail: kjunji@endmet.med.osaka-u.ac.jp

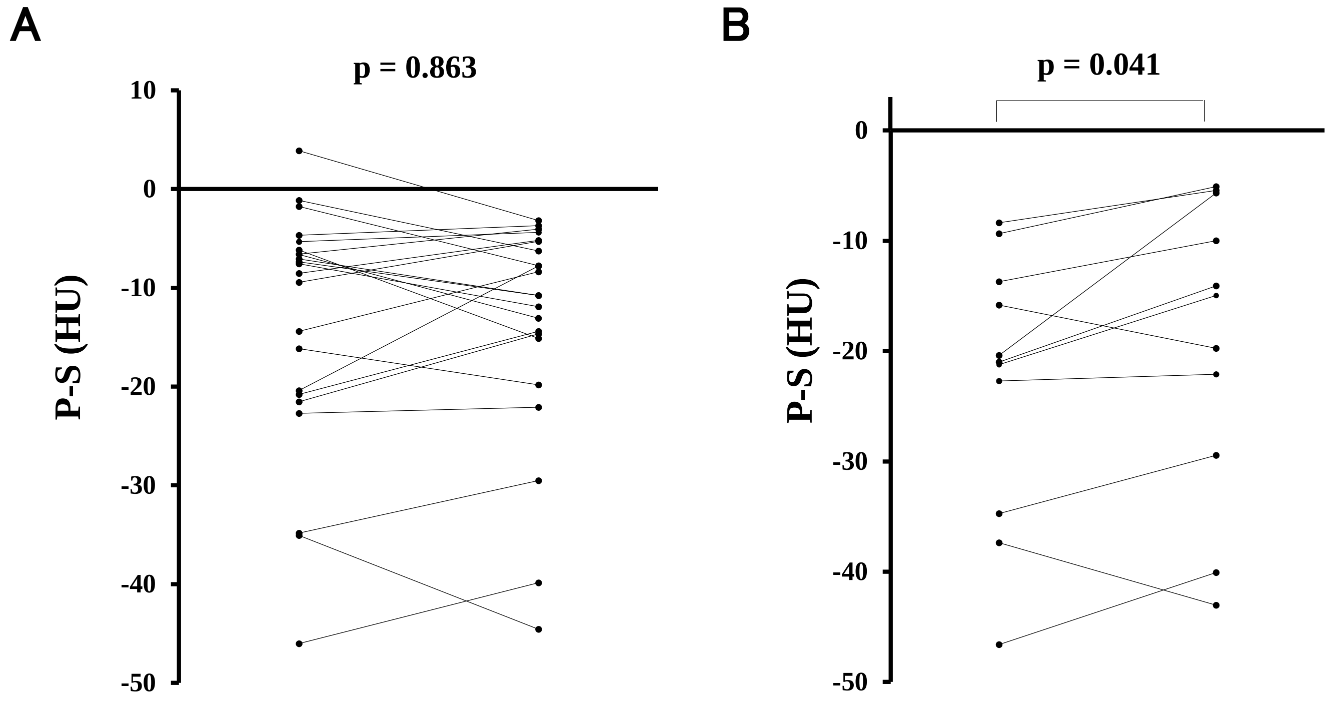

Supplemental Figure 1

Change in P-S values after treatment with SGLT-2 inhibitors in patients with type 2 diabetes. A: all patients (n = 22), B: the patients whose P-S values were less than -8 HU (n = 11).

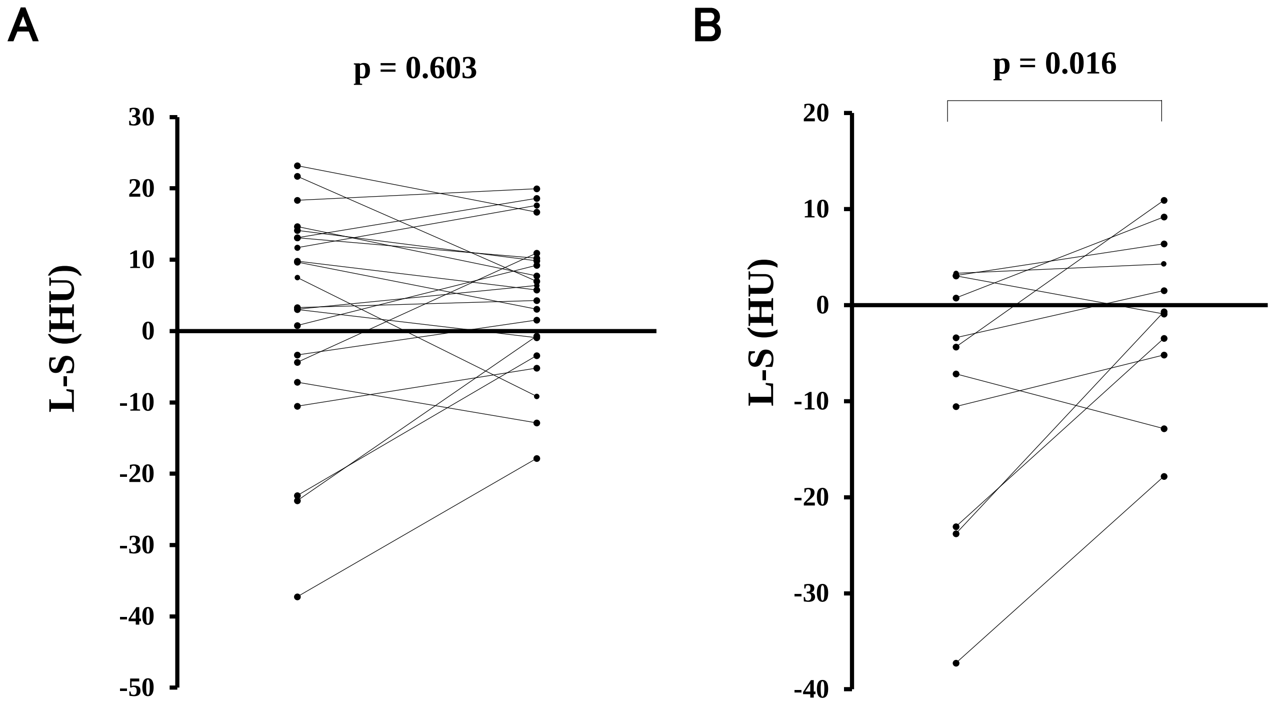

Supplemental Figure 2

Change in L-S values after treatment with SGLT-2 inhibitors in patients with type 2 diabetes. A: all patients (n = 22), B: the patients whose L-S values were less than 3.9 HU (n = 11).
